# Geographical Distribution of Pancreatic Cancer in the State of Mississippi by Incidence and Mortality From 2003 to 2019

**DOI:** 10.7759/cureus.31605

**Published:** 2022-11-17

**Authors:** Basil N Nduma, Solomon Ambe, Chukwuyem Ekhator, Ekokobe Fonkem

**Affiliations:** 1 Internal Medicine, Merit Health Wesley, Hattiesburg, USA; 2 Neurology, Baylor Scott & White Health, Mckinney, USA; 3 Neuro-Oncology, New York Institute of Technology, College of Osteopathic Medicine, Old Westbury, USA; 4 Neuro-Oncology, Baylor Scott and White Health, Temple, Temple, USA

**Keywords:** mortality rate, incidence rate, rural-urban divide, cancer coalition region, public health district, pancreatic cancer

## Abstract

Background: Pancreatic cancer can be a very debilitating disease. In the USA and around the world, pancreatic cancer is among the causes of cancer-related deaths. This study aims to highlight mortality and incidence rates of pancreatic cancer by geographic location.

Methods: The study area is the state of Mississippi with a targeted time period between 2003 and 2019. The Mississippi Cancer Registry is the source of data for this study. The subject under investigation was divided into two phases. The first phase involved analyzing data on the incidence rate while the second phase entailed data analysis of the pancreatic cancer mortality rate in Mississippi. In both phases, the focus was on three categories of geographic locations in Mississippi, which include public health districts, the regional cancer coalitions in the state, and the interplay between rural and urban locations. Descriptive and inferential statistical approaches with graphical techniques and tabulations were utilized in data presentation.

Results: The results of this study demonstrate there are variations in the incidence rates of pancreatic cancer by geographic location in Mississippi. In the data analysis of the Mississippi public health districts, the worst-hit areas include the rural communities in the rural-urban regional analysis, the Delta region among the cancer coalition regions, and the Central District (incidence rates) and North District (mortality rates).

Conclusion: In Mississippi, there is a need for aggressive community-based participation and education. This approach will help improve screening and early detection of pancreatic cancer. Healthcare intake should be boosted and geared toward a reduction in mortality rates. To minimize disparities that eventually lead to differences in disease incidence and mortality from different locations, legislative and non-legislative authorities should advocate for equitable distribution of healthcare resources. An understanding of the geographic distribution of pancreatic cancer in a state will aid in the designation of specific primary prevention measures targeted in the worst-hit communities.

## Introduction

Globally, the pancreatic cancer toll tends to be higher in more developed regions [1]. Whereas the key reasons explaining such a vast difference relative to pancreatic cancer-related mortality rates are yet to emerge clearly. Some of the aspects that have been attributed to the variations include a lack of appropriate cataloging, treatment, and diagnosis of cancer cases [1]. In 2018 alone, for instance, pancreatic cancer led to 432,242 new deaths globally. In the same year, about 458,918 new cases of the disease were reported. Following this trend, it is projected that about 355,317 new cases will occur annually until 2040 [1]. In recent years, there have been major scientific breakthroughs in early detection and treatment techniques for various cancers. However, even with these advancements, five-year survival rates for pancreatic cancer are still as low as 9%. The etiology of this disease is still poorly understood, however, several studies have demonstrated some trends from which some risk factors have been identified. Some of them include chronic pancreatitis, non-O blood group, *Helicobacter pylori* infection, family history and genetic factors, ethnicity, age, chronic alcoholism, and cigarette smoking [1].

Statistical outcomes in the US context are more worrying. For every 100,000 persons, the rate of new cases and deaths has been documented. Relative to age-adjusted rates and also as per the 2016-2020 deaths and 2015-2019 cases, the death rate in the United States (US) stands at 11.1 while the incidence is 13.3 annually in every 100,000 people, respectively [2]. Still, within the US context, scholarly outcomes suggest that close to 1.7% of men and women are likely to have a pancreatic cancer diagnosis at some point in their lifetime, with the findings documented relative to the 2017-2019 data [3]. In the year 2019, the prevalence of pancreatic cancer within the USA was about 89,248 [4]. An additional question that emerges at this point is how many people are likely to survive five years or more following a pancreatic cancer diagnosis. Worthy of note is the relative survival which reflects the estimated percentage of patients projected to survive cancer effects. As such, the risk of death and other causes are excluded. With large groups of people used to inform the survival statistics, predicting exactly what is likely to happen to an individual patient is not likely, due to the fact that no two patients can be alike in the entirety. There are also great variations in treatment and the way they respond to treatment [5]. Indeed, specific scholarly observations highlight that the five-year relative survival in the USA is 11.5% [6].

Estimations for 2022 also indicate that new cases of pancreatic cancer might stand at 62,210 with the number of deaths projected to be 49,830 [7]. Demographically, it is slightly higher in rate in men than women, but the general trend is that it increases with increasing age [8]. With a poor survival rate, pancreatic cancer-related deaths have seen the affected populations distributed similarly to persons diagnosed with the condition. Challenges with detecting the disease early have been avowed that the challenge explains the low average survival time [9]. The disease’s situation in the USA is even more worrying. Pancreatic cancer is the third leading cause of death, with the age-adjusted data for 2016-2020 deaths, the pancreatic cancer death rate stood at 11.1 in every 100,000 men and women [10].

Importantly, it is by keeping track of survival, deaths, and cases over time that the emerging trends could pave the way for scientists to understand the degree to which progress might have been realized in relation to the mitigation of pancreatic cancer and its effects, as well as discern areas in need of additional research to curb evolving challenges, including establishing better treatments and improving screening. With analyses conducted via statistical models, when new cases of pancreatic cancer are considered, it is notable that on average, age-adjusted rates have been increasing by 0.5% annually between 2010 and 2019 [9]. Regarding death rates, the increase in age-adjusted values has stood at 0.1% annually between 2011 and 2020 [10].

In Mississippi, pancreatic cancer is among the deadly diseases. Due to health disparities, most individuals fail to receive treatment. The socioeconomic composition of the region accounts for notable healthcare disparities [11]. Some of the databases that have been used to give insight into mortality from the disease, treatment, and case diagnoses include the National Cancer Institute (NCI), Surveillance Epidemiology and End Results (SEER) program, and the Mississippi Cancer Registry [12]. In the year 2006, Mississippi reported the highest national pancreatic cancer-related death rate, which is 12.7 in every 100,000 men and women. With further consideration of age-adjusted incidence for every county, the rate would be seen to range as high as 26.91 in every 100,000 men and women [13]. Of individuals dying from pancreatic cancer in Mississippi, 51% had treatment at the American College of Surgeons (ACS) Commission on Cancer (CoC) hospitals [14]. For the remaining 49%, their fate remained unknown. In the 51%, which is the group that had been tracked at CoC facilities in the state, compared to the National Cancer Database (NCDB) nationwide CoC data, no significant difference was observed in relation to factors of first treatment modalities, stage of diagnosis, and age distribution [15]. An emerging inference is that Mississippi continues to report a large number of pancreatic cancer patients whose treatment is yet to be known. This research focused on pancreatic cancer incidence and mortality by geographic region in Mississippi state, with the period focus being between 2003 and 2019. The motivation is to reveal insight into any disparities that could be existing among public health districts, cancer coalition regions, and rural-urban divide. In turn, the findings would probably pave way for stakeholders in the state’s healthcare industry to establish a pancreatic cancer care system poised to be responsive to any emerging needs while striving to foster comprehensiveness, inclusivity, and accessibility among the selected geographic regions, hence improved health-related quality of life among residents [16-18].

## Materials and methods

As mentioned in the preceding section, the research context is Mississippi state. The purpose of this study is to uncover pancreatic cancer incidence and mortality rates based on geographic location in Mississippi, targeting the trends between 2003 and 2019. The geographic locations centered on factors such as public health districts, cancer coalition regions, and the rural-urban divide in Mississippi. The main source of data is the Mississippi Cancer Registry. The registry houses population data collected from respondents of long-form census questionnaires, as well as those obtained from health administrative datasets arising from platforms for mortality, hospitalization, cancer, and ambulatory care. The registry offers information regarding aspects such as cancer mortality, all incidents of cancer, geographic locations that entail cancer coalition regions, cancer sites of the body such as a case in which pancreatic cancer belongs to the digestive system, and the period in terms of years of interest to the researcher. To ensure a comparative analysis, the navigation of the site entailed comparing rural with urban areas in terms of disease incidence and associated mortality in Mississippi. This study also analyzes the comparison of pancreatic cancer mortality and incidence in different cancer coalition regions in Mississippi state, and pancreatic cancer incidence and mortality among Public Health Districts in Mississippi.

The key indicator of the study is geographic location. The motivation is to discern how geographic location impacts pancreatic cancer mortality and incidence rate, as well as the implications for healthcare providers at the local, state, and federal levels. In this study, incidence refers to new pancreatic cancer cases arising between 2003 and 2019. The mortality rate implies pancreatic cancer deaths that occurred between 2003 and 2019 and were physician certified as a fatality in which the primary contributing factor was pancreatic cancer.

Descriptive statistics and inferential statistics are utilized in reporting the findings in this study. Descriptive statistics involved generating summaries relative to the arising data samples, presented through the graphical and tabulation methods. On the other hand, inferential statistics involved drawing conclusions after examining the samples and outcomes presented by descriptive statistical approaches. Hence, inferential statistics were incorporated to complement descriptive statistics while seeking to make informed generalizations and conclusions about the subject being investigated, translating into the provision of recommendations as required, as well as the depiction of the implications for the healthcare system in Mississippi.

## Results

Incidence of pancreatic cancer by geographic location

**Figure 1:**
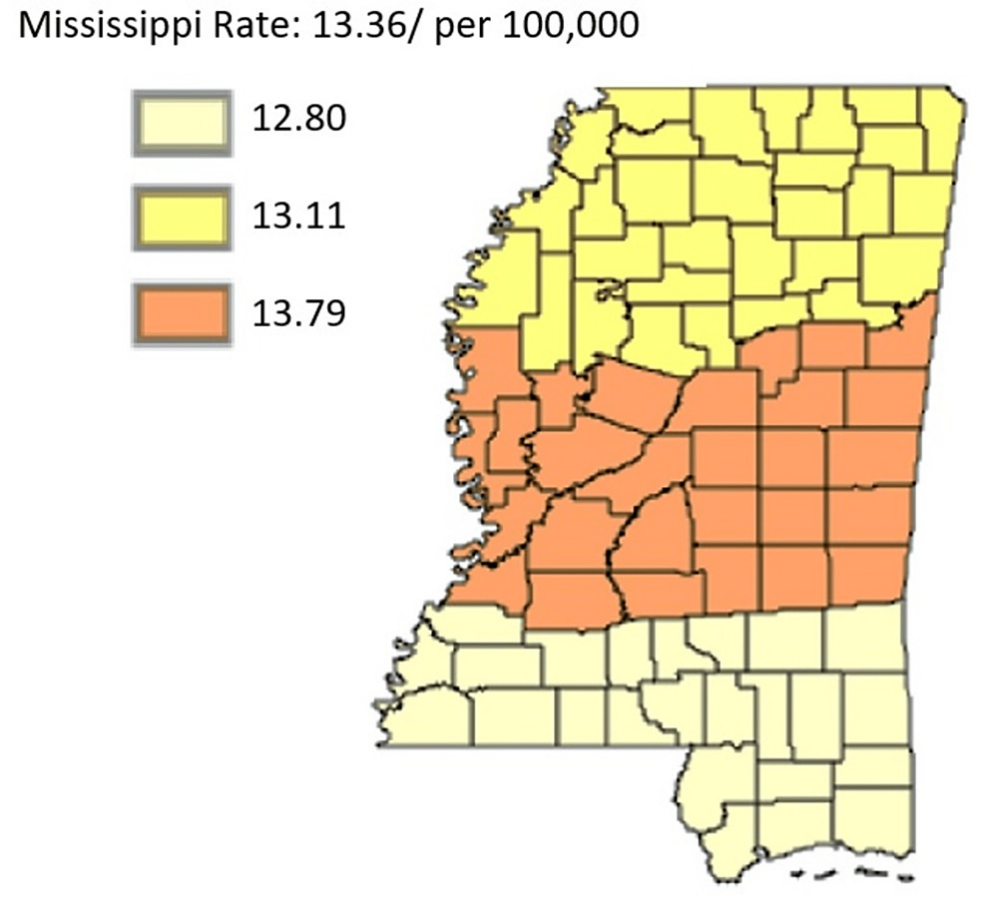
Pancreatic Cancer Incidence rate by Public Health Districts in Mississippi state (All genders and races) from 2003 to 2019 Age-adjusted to the 2000 US Standard Million Population. The population estimates for 2005 are adjusted to account for population shifts due to Hurricane Katrina (www.seer.cancer.gov/popdata/)

The focus of this section is on all cancer incidents, specifically all incidents of pancreatic cancer. The intention is to highlight differences among public health districts, cancer coalition regions, and between rural and urban areas as highlighted in Figure 1. The focus is also on all ethnic or racial groups, as well as all genders.

Incidence by public health district

The three public health districts include Central District, North District, and South District.

**Figure 2:**
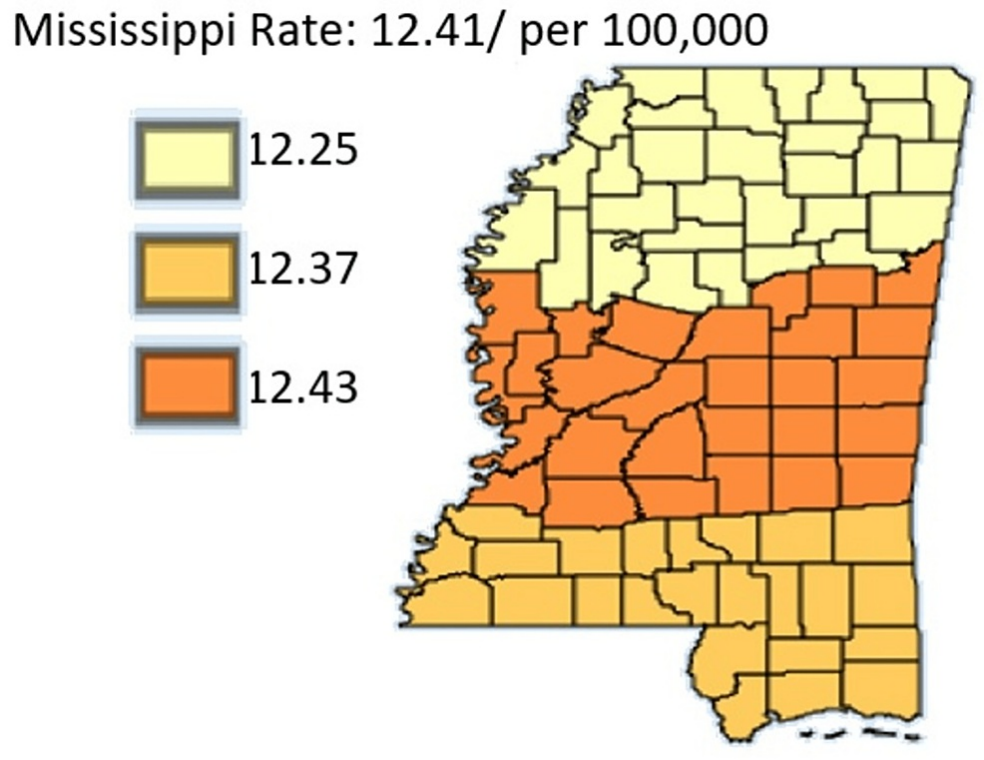
Pancreatic Cancer Incidence rate by Public Health Districts in Mississippi state in White residents only Age-adjusted to the 2000 US Standard Million Population. The population estimates for 2005 are adjusted to account for population shifts due to Hurricane Katrina (www.seer.cancer.gov/popdata/)

Figure 2 shows the pancreatic cancer incidence rate by Public Health Districts in Mississippi state in White residents only.

**Figure 3:**
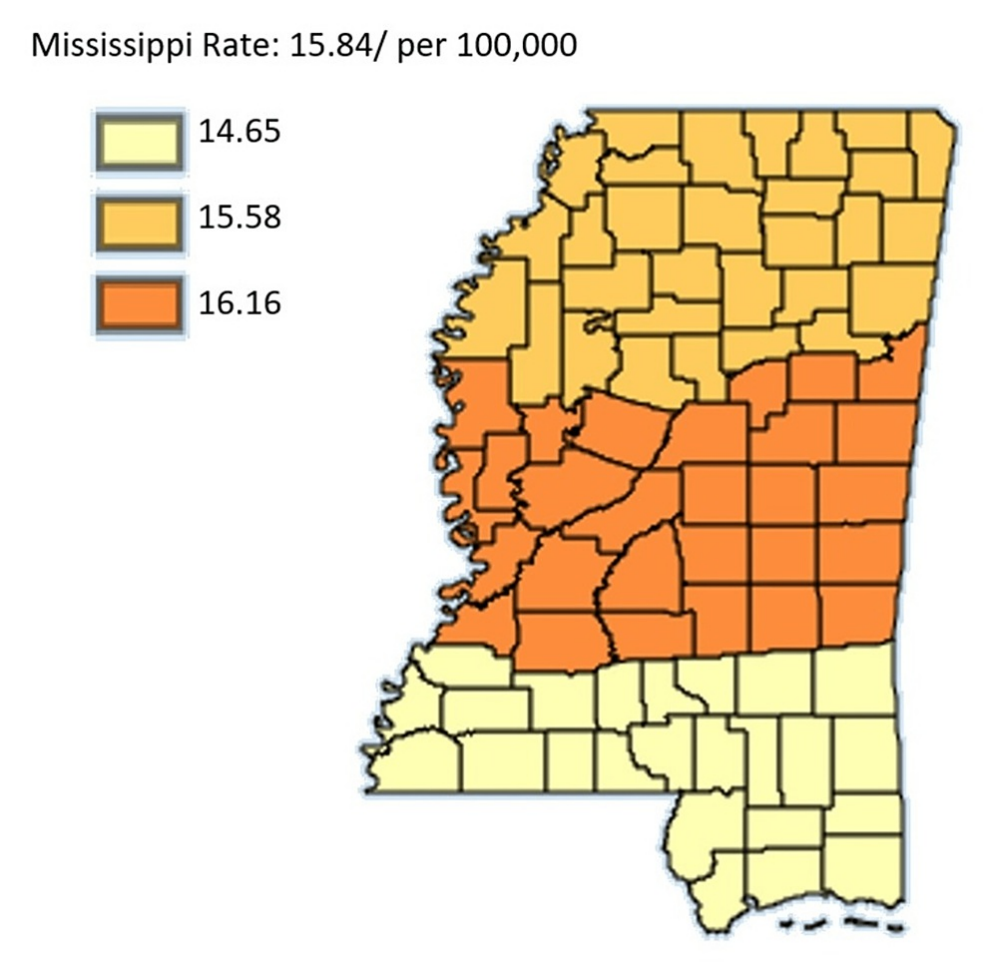
Pancreatic Cancer Incidence rate by Public Health Districts in Mississippi state in Black Residents only Age-adjusted to the 2000 US Standard Million Population. The population estimates for 2005 are adjusted to account for population shifts due to Hurricane Katrina (www.seer.cancer.gov/popdata/)

 Figure 3 shows the pancreatic cancer incidence rate by Public Health Districts in Mississippi state in Black residents only.

**Figure 4:**
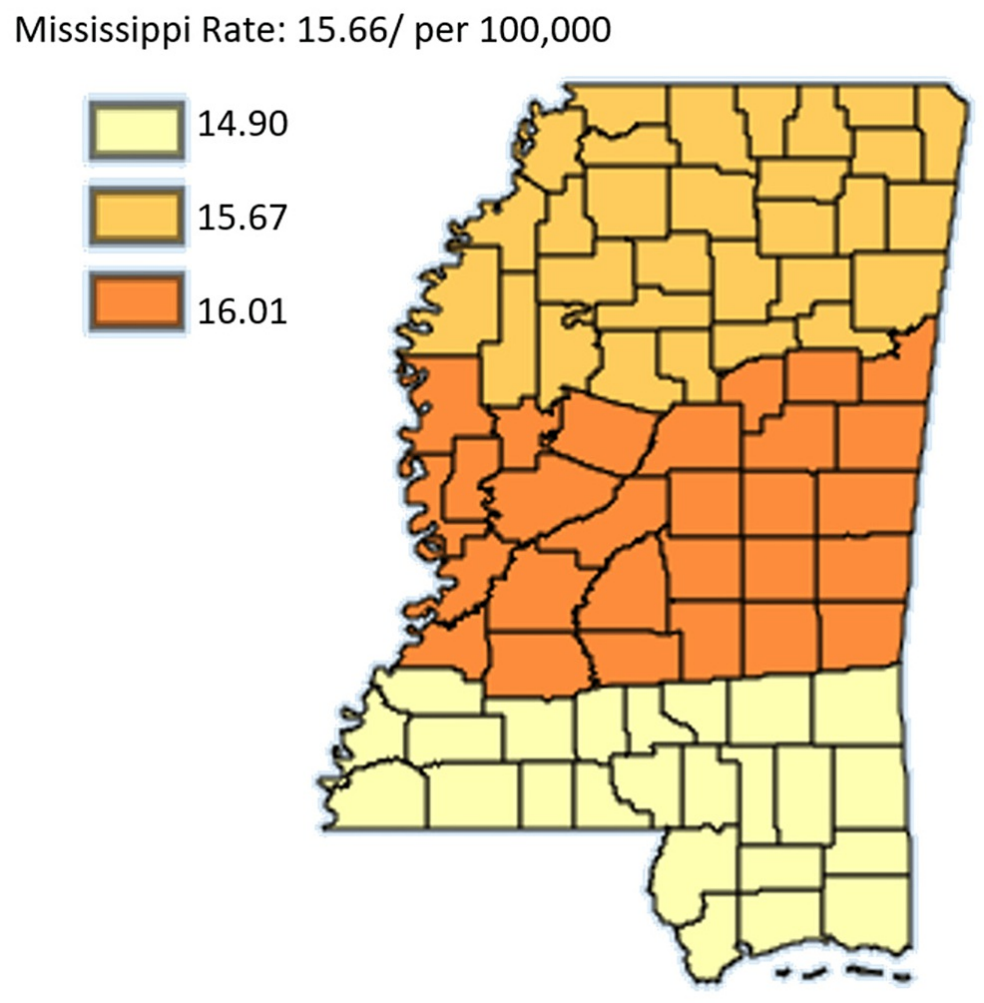
Pancreatic Cancer Incidence rate by Public Health Districts in Mississippi state in male residents only Age-adjusted to the 2000 US Standard Million Population. The population estimates for 2005 are adjusted to account for population shifts due to Hurricane Katrina (www.seer.cancer.gov/popdata/)

Figure 4 shows the pancreatic cancer incidence rate by Public Health Districts in Mississippi state in male residents only.

**Figure 5:**
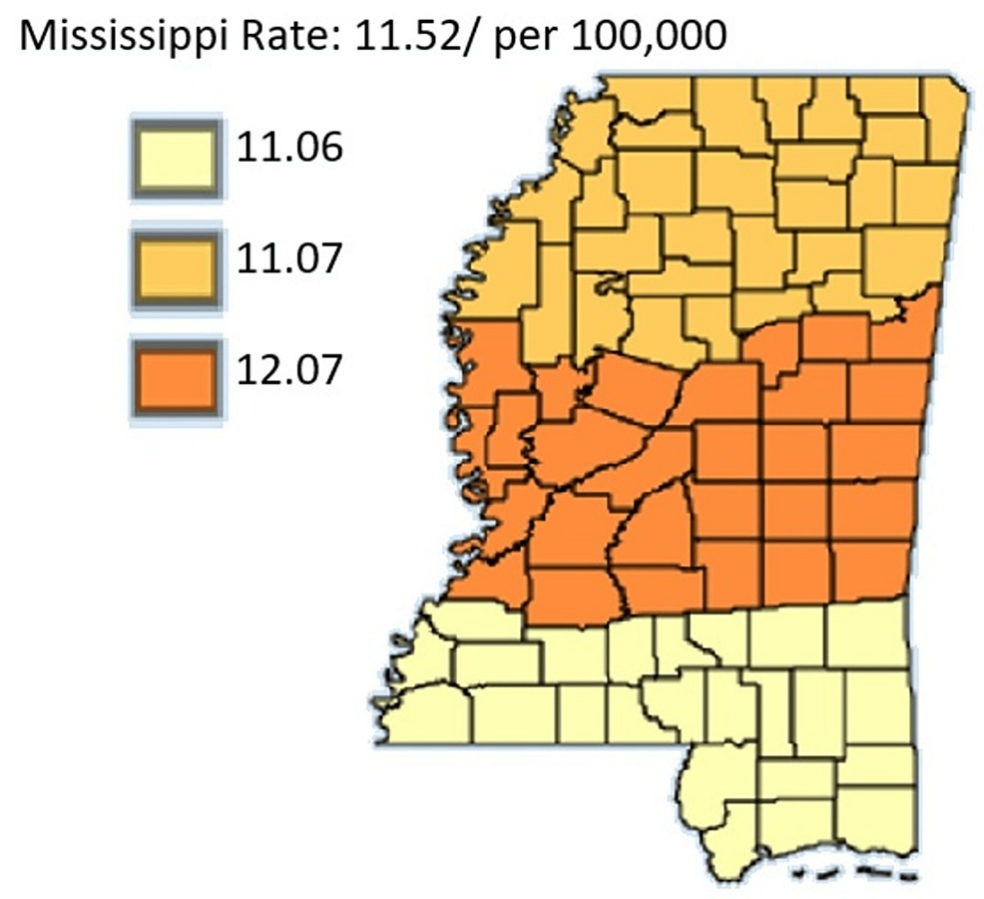
Pancreatic Cancer Incidence rate by Public Health Districts in Mississippi state in female residents only Age-adjusted to the 2000 US Standard Million Population. The population estimates for 2005 are adjusted to account for population shifts due to Hurricane Katrina (www.seer.cancer.gov/popdata/)

Figure 5 shows the pancreatic cancer incidence rate by Public Health Districts in Mississippi state in female residents only.

As outlined in Figure 1, pancreatic cancer incidence rate is by Public Health Districts in Mississippi state in all genders and races. Based on Figure 1, the age-adjusted rate was highest in the Central District, followed by the North District and the South District, respectively.

In Figures 2-3, the age-adjusted incidence rate of pancreatic cancer is highest among Black residents compared to White residents. Highest rates among Black residents was in the Central District with a rate of 16.16 and lowest in the Southern Region with rate of 14.65. For White residents, their highest incidence was in the Central District as they recorded 12.43. The lowest incidence for White residents was in the Northern District at a rate of 12.25.

**Figure 6:**
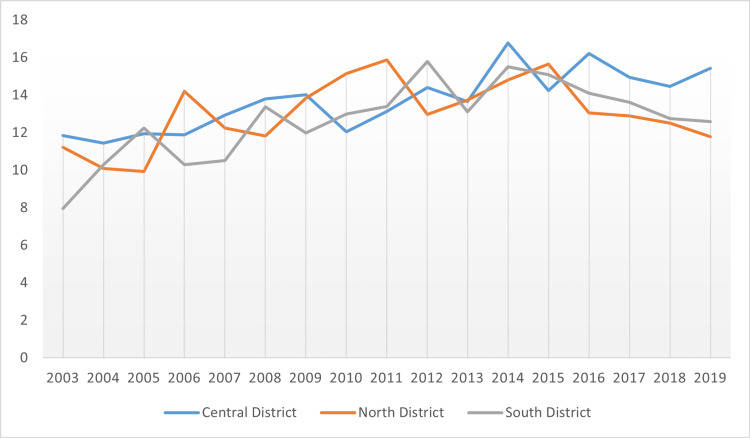
Incidence of pancreatic cancer per Public Health Districts’ comparison in all races and genders from 2003 to 2019

In Figures 4-5, the age-adjusted incidence rate of pancreatic cancer was higher among the male residents compared to the female residents. Males recorded their highest incidence rate in the Central District which was 16.01 and the lowest incidence in the Southern District which was 14.90. Female residents likewise recorded their highest incidence rate in the Central District of 12.07 and their lowest incidence in the Southern District of 11.06. Figure 6 offers a comparative analysis of the three public health districts between 2003 and 2019.

**Figure 7:**
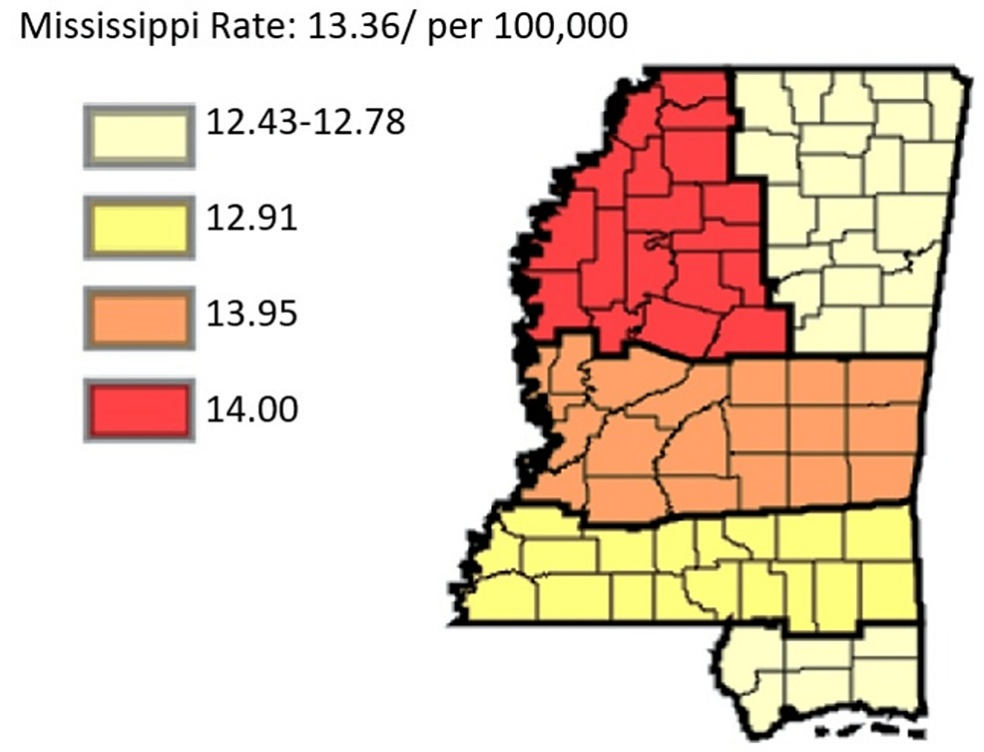
Cancer Coalition Regions’ pancreatic cancer overview Age-adjusted to the 2000 US Standard Million Population. The population estimates for 2005 are adjusted to account for population shifts due to Hurricane Katrina (www.seer.cancer.gov/popdata/)

**Figure 8:**
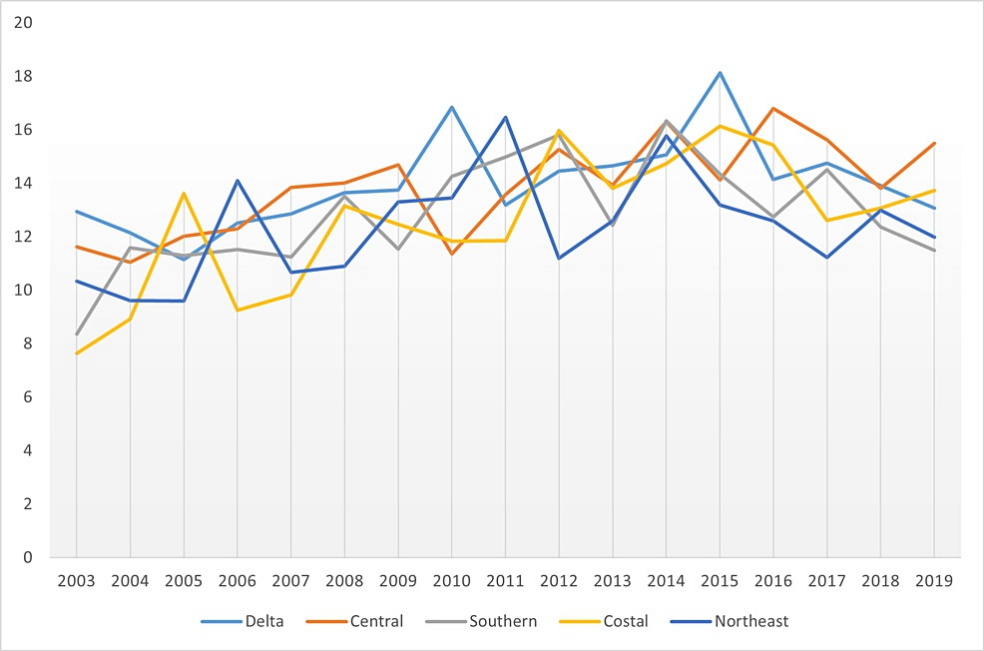
Comparison of incidence of pancreatic cancer in Cancer Regional Coalitions

Next, with the factor of pancreatic cancer incidence still the focus, the study proceeded to offer a comparative analysis among cancer regional coalitions. The coalitions include Delta, Central, Southern Coastal, and Northeast. The results obtained were as follows in Figures 7-8.

Indeed, Delta exhibited the highest rates, with the average age-adjusted rate standing at 14.00. The incidence then proceeded to stand in other regional coalitions at 13.95, 12.91, 12.78, and 12.43 for the cases of Central, Southern, Coastal, and Northeast Regional Coalition, respectively. Figure 8 gives a more in-depth insight into the specific incidents for each year.

**Figure 9:**
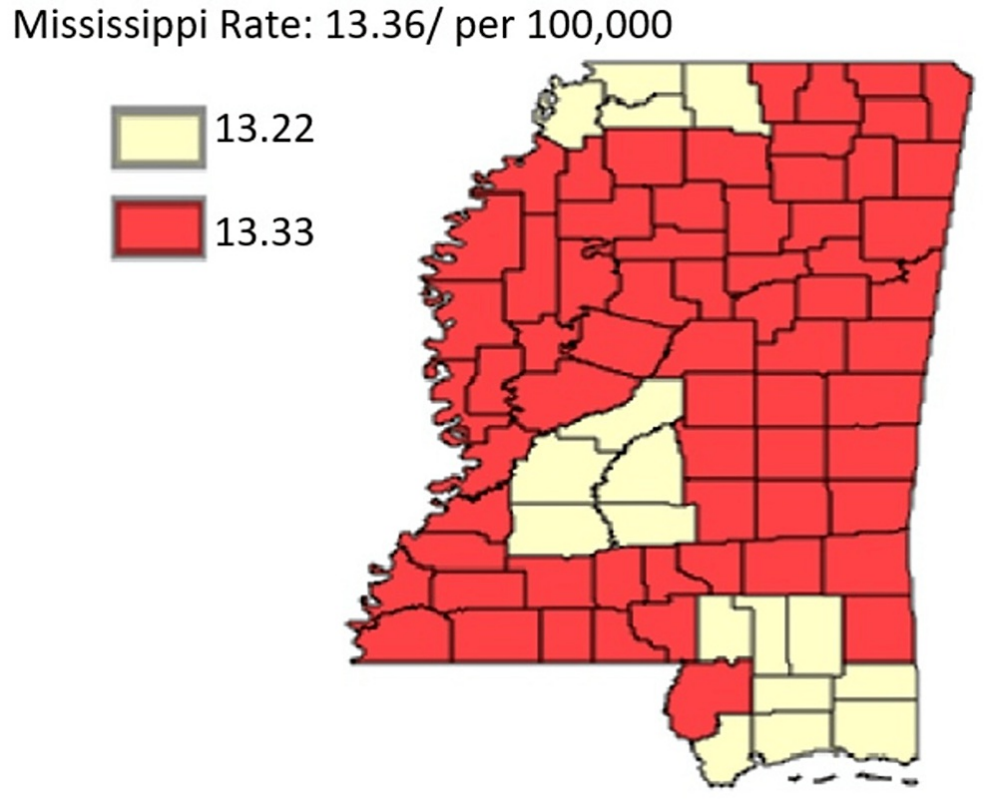
The rural-urban divide in pancreatic cancer incidence Age-adjusted to the 2000 US Standard Million Population. The population estimates for 2005 are adjusted to account for population shifts due to Hurricane Katrina (www.seer.cancer.gov/popdata/)

**Figure 10:**
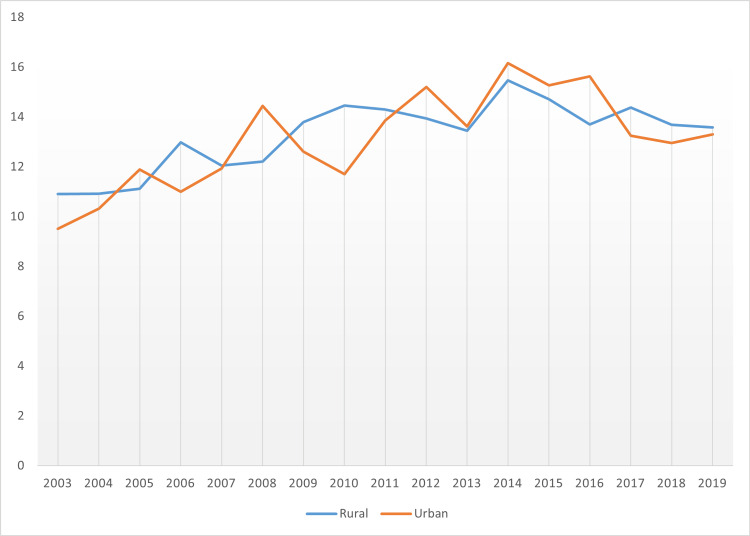
A comparative analysis of rural versus urban pancreatic cancer incidence

Finally, the assessment of the incidence of pancreatic cancer by geographic location was focused on by rural-urban divide as outlined in Figures 9-10. Hence, there was the presentation of data regarding the incidence of pancreatic cancer in Mississippi state’s rural areas between 2003 and 2019, alongside data in the same time period for the case of urban zones, aiming to provide an informed comparative analysis.

Figure 9 shows rural areas rated worse compared to urban zones. Thus, it was inferred that the incidence was higher in rural areas, standing at an average age-adjusted rate of 13.33 compared to the average age-adjusted rate for urban areas, which was 13.22. The figure offers more insight into the respective years’ breakdown data depicting a divide in the incidence rate between rural and urban regions.

The general trend was that both regions exhibited notable fluctuations. In most cases, years, when the incidence rate was higher in rural zones coincided with lower rates in the urban zones and vice versa, except for the years 2007 and 2013 when there was near parity in the rates for both regions.

Mortality rate from pancreatic cancer by geographic location

**Figure 11:**
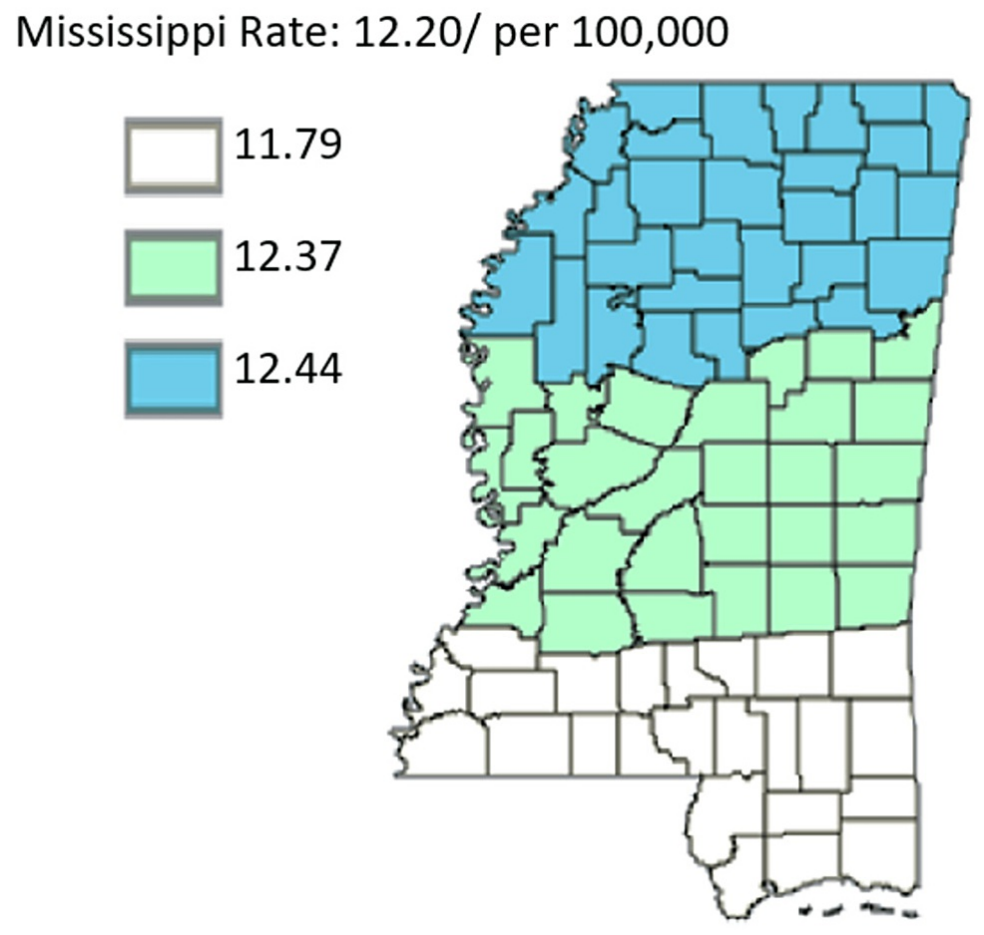
Age-adjusted mortality rates of pancreatic cancer in Mississippi by public health district from 2003 to 2019 with all genders included Age-adjusted to the 2000 US Standard Million Population. The population estimates for 2005 are adjusted to account for population shifts due to Hurricane Katrina (www.seer.cancer.gov/popdata/)

In this study, with geography being the central subject under investigation, the second main theme that was being investigated entails probable variations in the mortality rate based on three broad factors of public health districts, cancer coalition regions, and rural and urban areas. The motivation for investigating this second theme was to give insight into any variations in the rates between or among regions, as well as shed light on probable features accounting for the perceived fluctuations in mortality rates per region, eventually giving insight into the needed actions by healthcare authorities in Mississippi. Figure 11 outlines the age-adjusted mortality rates of pancreatic cancer in Mississippi by public health district from 2003 to 2019 in all genders.

**Figure 12:**
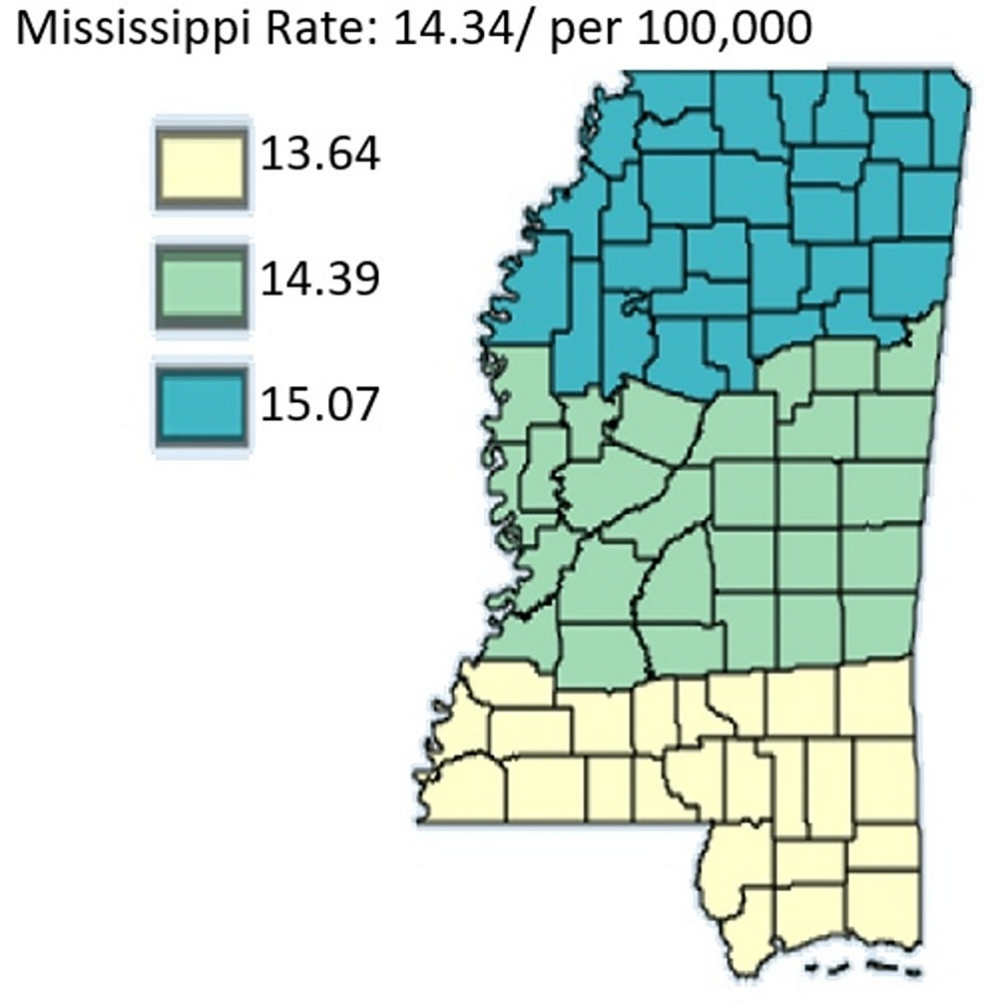
Age-adjusted mortality rates of pancreatic cancer in Mississippi by public health district from 2003 to 2019 in males only Age-adjusted to the 2000 US Standard Million Population. The population estimates for 2005 are adjusted to account for population shifts due to Hurricane Katrina (www.seer.cancer.gov/popdata/)

Figure 12 outlines the age-adjusted mortality rates of pancreatic cancer in Mississippi by public health district from 2003 to 2019 in males only.

**Figure 13:**
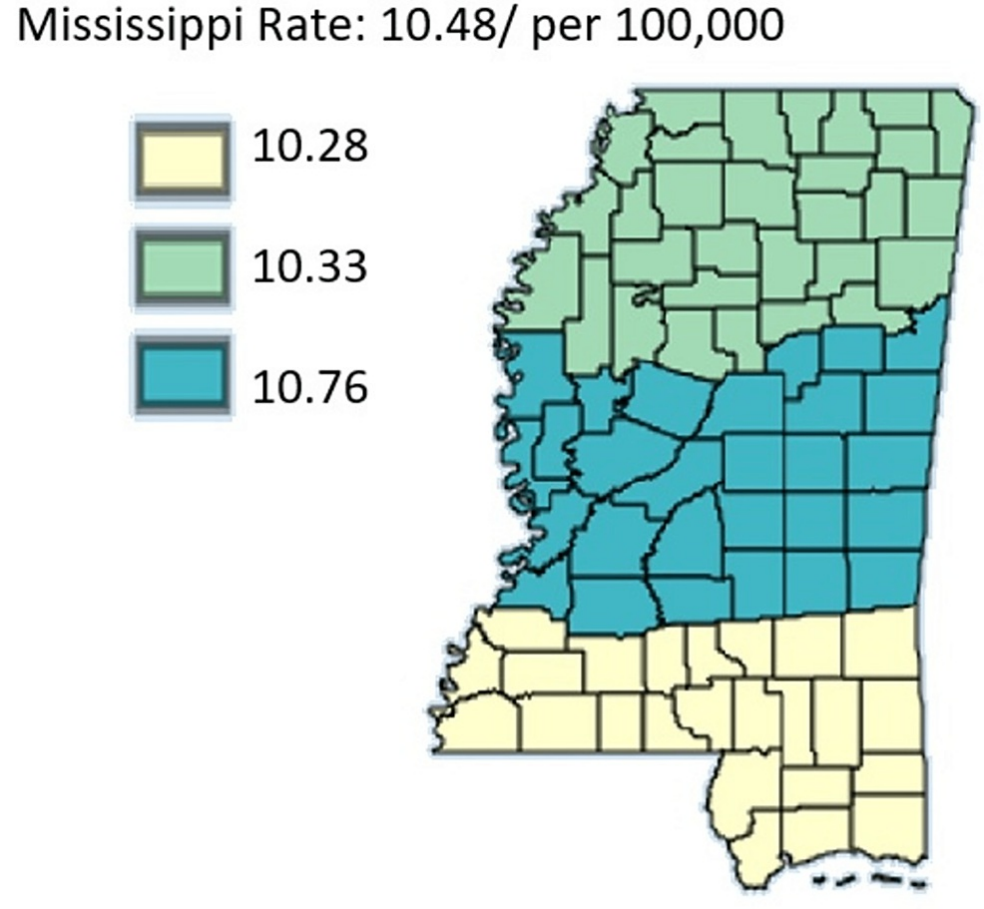
Age-adjusted mortality rates of pancreatic cancer in Mississippi by public health district from 2003 to 2019 in females only Age-adjusted to the 2000 US Standard Million Population. The population estimates for 2005 are adjusted to account for population shifts due to Hurricane Katrina (www.seer.cancer.gov/popdata/)

Figure 13 shows the age-adjusted mortality rates of pancreatic cancer in Mississippi by public health district from 2003 to 2019 in females only.

**Figure 14:**
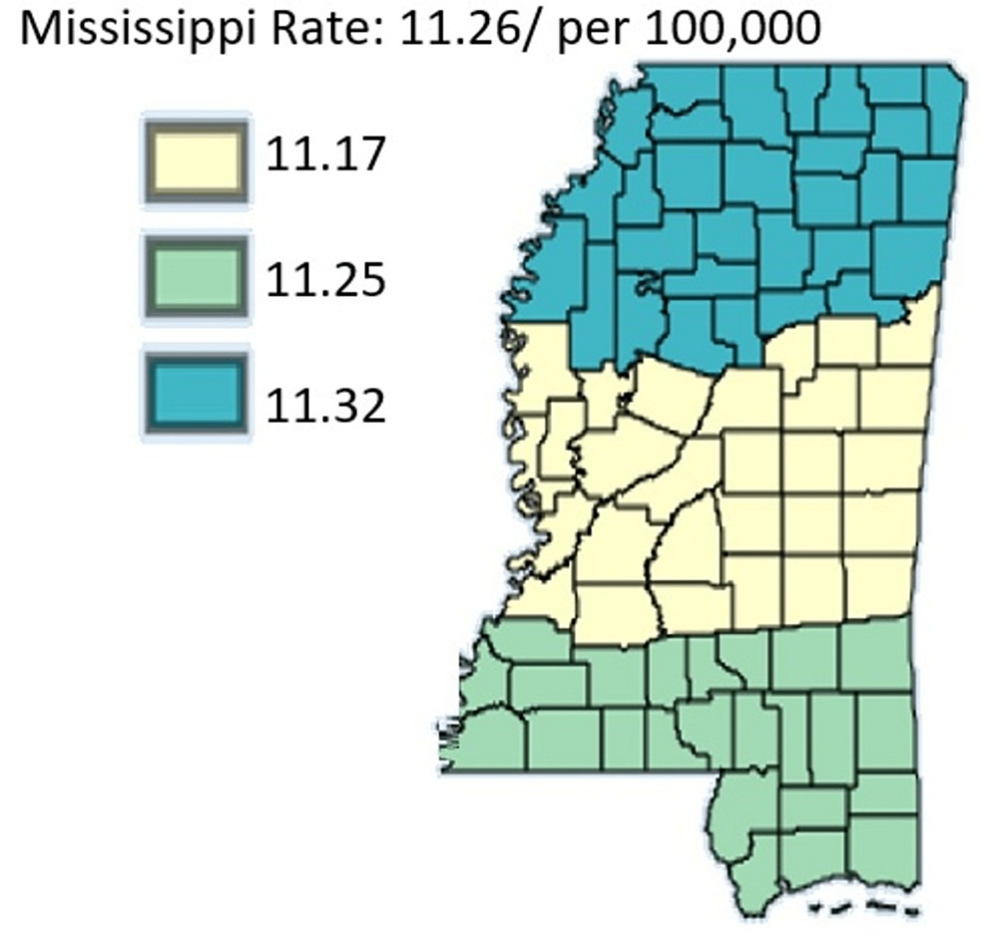
Age-adjusted mortality rates of pancreatic cancer in Mississippi by public health district from 2003 to 2019 in White residents only Age-adjusted to the 2000 US Standard Million Population. The population estimates for 2005 are adjusted to account for population shifts due to Hurricane Katrina (www.seer.cancer.gov/popdata/)

Figure 14 shows the age-adjusted mortality rates of pancreatic cancer in Mississippi by public health district from 2003 to 2019 in White residents only.

**Figure 15:**
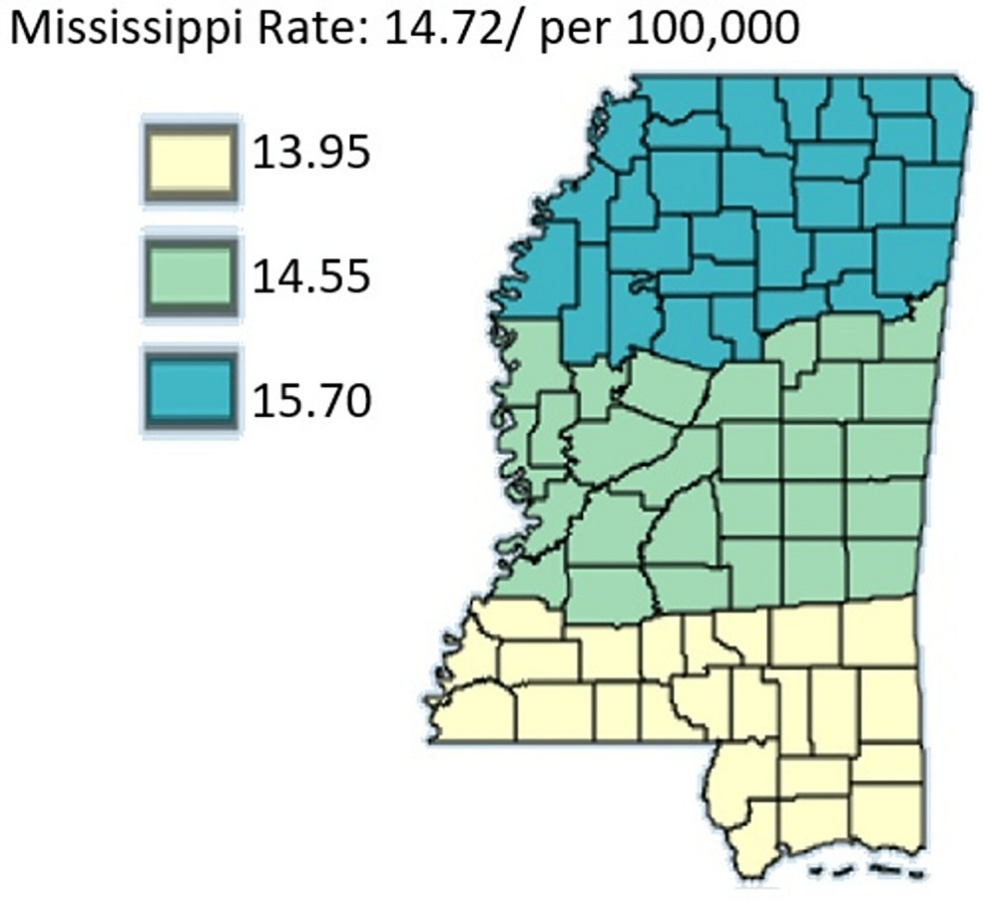
Age-adjusted mortality rates of pancreatic cancer in Mississippi by public health district from 2003 to 2019 in Black residents only Age-adjusted to the 2000 US Standard Million Population. The population estimates for 2005 are adjusted to account for population shifts due to Hurricane Katrina (www.seer.cancer.gov/popdata/)

Figure 15 shows the age-adjusted mortality rates of pancreatic cancer in Mississippi by public health district from 2003 to 2019 in Black residents only.

In Figure 11, it can be noted that the North District reported the highest age-adjusted rate of 12.44, followed by the Central District and the South District, whose age-adjusted rates were 12.37 and 11.79, respectively. The figure offers comparative outcomes for each year.

Figures 12-13, which compare mortality rates between males and females, males overall had significantly higher incidence throughout the state. Males in the Northern District were most hit with rates of 15.07 while males in the Southern District were least hit by pancreatic cancer with rates at 13.64. Females on the other hand lower mortality across the state with the females in the Central District having the highest mortality rate of 10.76 compared to women in the Southern District who had the least mortality in the state at 10.28. Overall, the map for mortality among males mirrors the mortality of the entire state which further confirms that the mortality rate of pancreatic cancer in males in Mississippi actually drives the trend of mortality of pancreatic cancer in the state.

**Figure 16:**
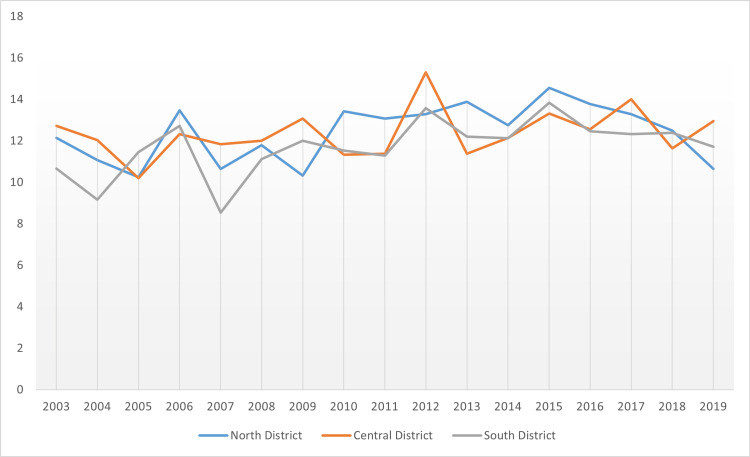
Comparative outcomes of pancreatic cancer mortality by public health district from 2003 to 2019

Figures 14-15 compare the mortality rate of pancreatic cancer among White and Black residents, it can be seen that Black residents have mortality rates compared to White residents. The highest incidence rate for Black residents was in the Northern District which had a mortality rate of 15.70 and the lowest incidence for Black residents was in the Southern District which was 13.95. For White residents, the mortality rate was highest in the Northern District at 11.32 and lowest in the Central District at 11.17. Notably, the incidence rate as per Figure 16 depicted that the incidence rate was highest in the Central District, followed by the North District, and lastly the South District.

**Figure 17:**
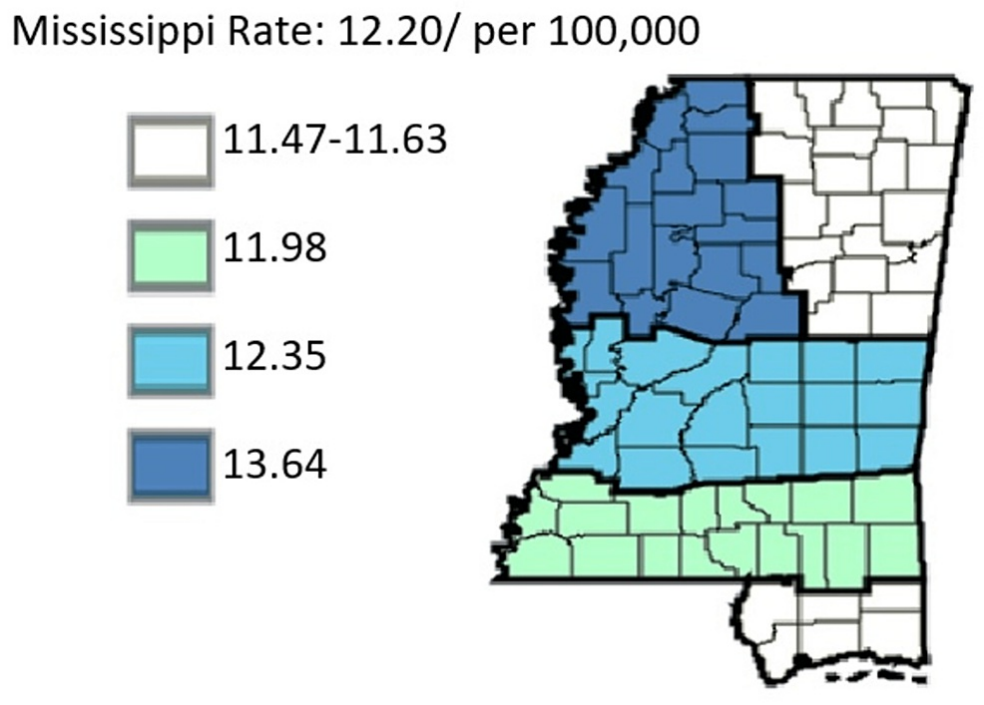
An overview of pancreatic cancer mortality by the cancer regional coalition Age-adjusted to the 2000 US Standard Million Population. The population estimates for 2005 are adjusted to account for population shifts due to Hurricane Katrina (www.seer.cancer.gov/popdata/)

On mortality, however, the highest rate was in the North District, followed by the Central District, and finally the South District. The only consistent trend, therefore, was that it was only in the South District that the incidence rate was lowest, with the mortality rate also proving lowest in the same public health district. Next, this study offered a comparative analysis of pancreatic cancer-related mortality rate by the cancer regional coalition in Mississippi state. Figure 17 shows an overview of pancreatic cancer mortality by the cancer regional coalition.

**Figure 18:**
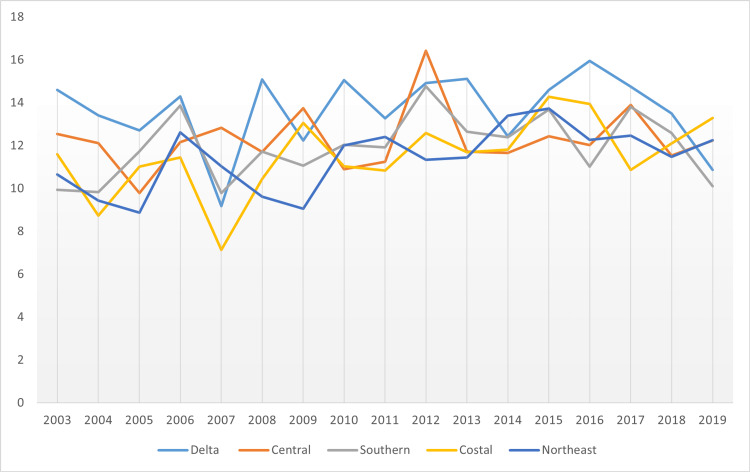
Comparative mortality rate outcomes of pancreatic cancer for coalition regions from 2003 to 2019

Indeed, there was a similarity in trends between cancer incidence and cancer mortality. From Figure 8, the incidence rate was highest in Delta Regional Coalition, followed by Central, Southern, Coastal, and Northeast regional coalitions. In Figure 17, the mortality rates assumed the same trend, with Delta leading in times of cancer mortality rates, followed by Central, Southern, Coastal, and Northeast regional coalitions in that order. Hence, it was inferred that in Mississippi, between 2003 and 2019, coalition regions that had the highest incidence rate also had the highest mortality rates. Conversely, coalition regions with the lowest incidence rates also depicted the lowest mortality rates. Figure 18 shows the comparative mortality rate outcomes of pancreatic cancer for coalition regions from 2003 to 2019.

**Figure 19:**
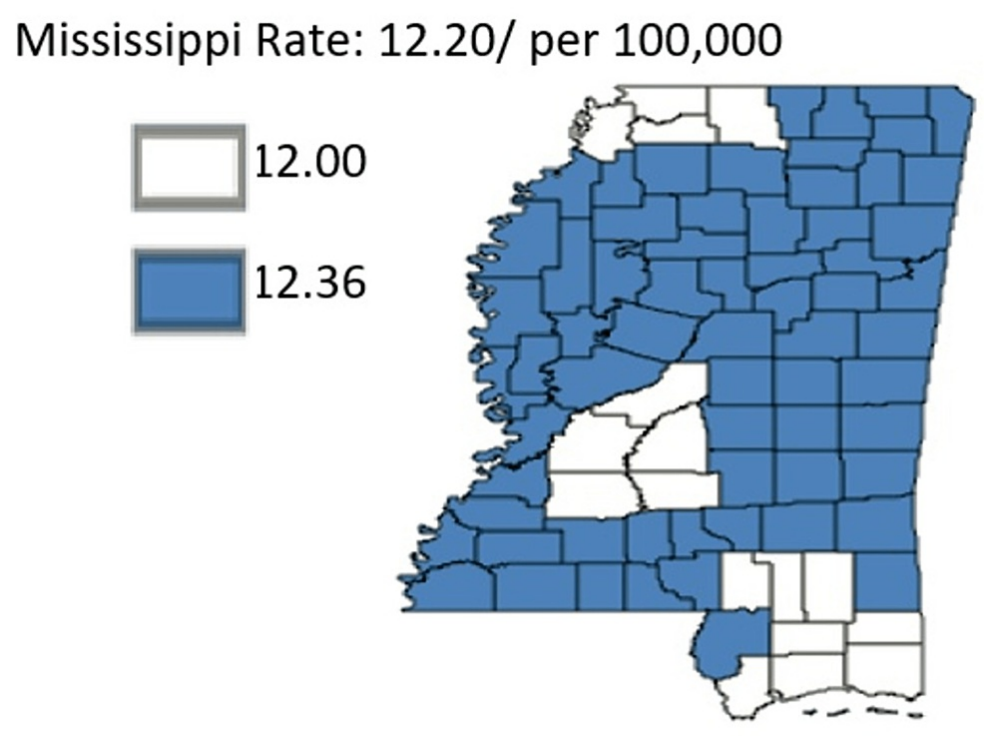
An overview of pancreatic cancer mortality rural-urban divide of Mississippi state Age-adjusted to the 2000 US Standard Million Population. The population estimates for 2005 are adjusted to account for population shifts due to Hurricane Katrina (www.seer.cancer.gov/popdata/)

Figure 19 shows an overview of pancreatic cancer mortality in the rural-urban divide.

**Figure 20:**
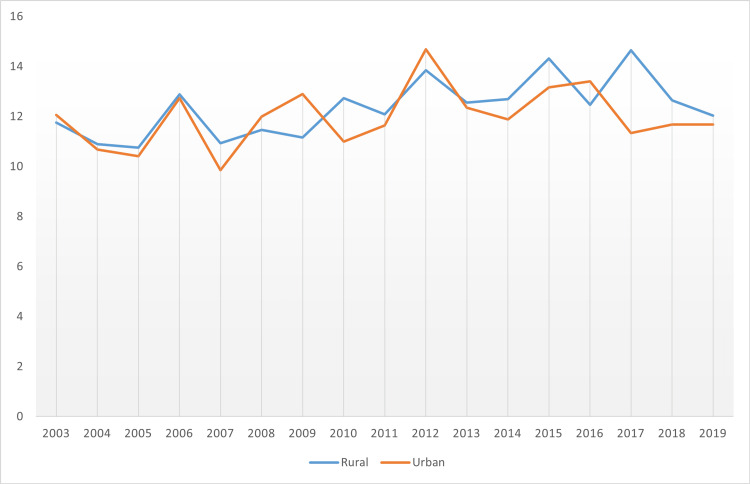
Pancreatic cancer mortality rate in the rural versus urban divide in Mississippi state

Similar to the theme of incidence rate, the mortality rate factor saw rural areas fare worse than urban regions. More specific data for the average age-adjusted data in the respective years is provided in Figure 20.

Despite rural areas being worse hit regarding mortality rate due to pancreatic cancer, it was notable that there was a larger number of years when there was near parity. These years included 2003, 2004, 2005, 2006, 2011, and 2013. Some factors that affect pancreatic cancer incidence and mortality include smoking habits which have been reported in studies to double the incidence of pancreatic cancer, diet, and obesity, whereby regular use of foods high in fat reflects a risk factor for the condition, chronic, heavy use of alcohol, genetics and family history [19-25].

## Discussion

From the results, the highest incidence and mortality rates regarding coalition regions or regional coalitions were reported in Delta, with the Northeast reporting the lowest mortality rate and incidence. On the rural-urban divide, rural areas fared worse than urban regions. Lastly, South District experienced the lowest incidence rates and mortality rates. In the same period, with the average age-adjusted rates on the focus, the highest incidence rate in relation to the factor of public health districts was reported in Central District while the highest mortality rate among the public health districts was reported in North District. Also of note is that in all geographic regions, male residents consistently had higher incidence and mortality rates compared to female residents and Black residents consistently had higher incidence and mortality rates compared to White residents. Important to note, however, is that two factors play a moderating role relative to the outcomes. First, the population is not distributed uniformly between and among the selected geographic locations. There are significantly many more rural communities than urban communities in Mississippi. In addition, the data available for this study was age-adjusted, thus data on the incidence and mortality of pancreatic cancer for specific age groups in the population was not available to be used for this study. Whether these factors might explain why more incidence and mortality rates were reported in rural locations compared to urban zones remains at stake [18].

Despite the aforementioned, the study’s point of convergence with previous scholarly observations include variations in the rates from one region or location to another, gender differences in incidence and mortality whereby men had higher incidence and mortality of pancreatic than women, and also race or ethnicity whereby Black residents had significantly higher incidence and mortality rates [18].

From the outcome of this study, the implication for Mississippi is that, with the high pancreatic cancer prevalence in the state, alongside incidence and mortality rates that vary from one geographic location to another, certain mitigation measures are worthy. In particular, primary prevention is of much importance because screening recommendations for the condition are yet to be established. stakeholders in the state’s healthcare sector ought to understand these geographic variations in pancreatic cancer, as well as the specific risk factors in the highest-hit region, so that specific primary prevention might be realized. Some potentially modifiable risk factors per scholastic literature include diet, diabetes mellitus, tobacco smoking, obesity, and chronic alcohol abuse. The best preventive strategy for this condition has been affirmed to entail risk reduction via lifestyle modification [23-25]. Lifestyle modification includes regular exercise, a diet high in fruits and vegetables, a healthy weight, and smoking cessation [26]. In addition, this study also recognizes a possible genetic component in the incidence and mortality of pancreatic cancer evident by higher incidence rates in the Black population as well as higher incidence in male residents compared to female residents. The state can enact legislations that will encourage further research into this possible genetic component of pancreatic cancer.

As a limitation of this study, some factors that affect pancreatic cancer incidence and mortality in the state of Mississippi could not be assessed due to limited data availability. These factors include smoking habits, diet, obesity, alcohol use, genetics, and family history. Although the effects of these factors on pancreatic cancer have been widely published in the literature, more studies are needed to extrapolate how these factors play a role in the incidence and mortality trends in the state of Mississippi. The results of this study demonstrated that there are variations in disease mortality rate and incidence by geographic location in Mississippi. Worst-hit areas include rural zones in relation to the factor of the rural-urban divide, Delta in relation to the factor of cancer coalition regions, and Central District (incidence rates) and North District (mortality rates) in relation to the factor of public health districts. Also of note is that in all geographic regions, male residents consistently had higher incidence and mortality rates compared to female residents and Black residents consistently had higher incidence and mortality rates compared to White residents.

## Conclusions

Understanding the geographic distribution and trends in incidence and mortality rates of pancreatic cancers lays a framework for various target interventions. There is a need for aggressive community-based participatory processes and education to address the needs of under-screened groups and improvement in healthcare intake to reduce the mortality rate of pancreatic cancer. To ensure parity in service provision as a way of mitigating burdens in the worst-hit geographic regions, state authorities need to foster equitable healthcare resource distribution as well as understand the geographic distribution of pancreatic cancer in the state to design specific location-targeted primary prevention measures in the worse hit communities.
